# The effect of Farnesoid X receptor agonist tropifexor on liver damage in rats with experimental obstructive jaundice

**DOI:** 10.1590/ACB360902

**Published:** 2021-10-25

**Authors:** Huseyin Kilavuz, Umit Turan, Atilla Yoldas, Fatma Inanc Tolun, Burak Tanriverdi, Asli Yaylali, Abit Yaman, Mehmet Kemal Yener, Oktay Irkorucu

**Affiliations:** 1 MD. Department of General Surgery - Basaksehir Cam and Sakura City Hospital - Istanbul, Turkey.; 2 MD. Department of General Surgery - Adana City Research and Training Center - Saglik Bilimleri University - Adana, Turkey.; 3 MD. Department of Anatomy - School of Medicine - Kahramanmaras Sutcu Imam University - Kahramanmaras, Turkey.; 4 MD. Department of Biochemistry - School of Medicine - Kahramanmaras Sutcu Imam University - Kahramanmaras, Turkey; 5 MD. Department of Histology and Embriyology and IVF Center - School of Medicine - Kahramanmaras Sutcu Imam University - Kahramanmaras, Turkey.; 6 MD. Department of General Surgery - Ceylanpinar Public Hospital - Sanliurfa, Turkey.; 7 MD. University of Sharjah - College of Medicine - Clinical Sciences Department – Sharjah, UAE.

**Keywords:** Cholestasis, Liver, Jaundice, Ursodeoxycholic acid, Rats

## Abstract

**Purpose:**

To investigate experimentally the effects of Tropifexor, a farnesoid X receptor agonist, on liver injury in rats with obstructive jaundice.

**Methods:**

Forty healthy Wistar albino female rats were divided randomly in selected groups. These groups were the sham group, control group, vehicle solution group, Ursodeoxycholic acid group and Tropifexor group. Experimental obstructive jaundice was created in all groups, except the sham one. In the blood samples obtained, aspartate transaminase (AST), alanine transaminase (ALT), alkaline phosphatase (ALP), gamma-glutamyl transferase (GGT), total bilirubin and direct bilirubin levels were established and recorded. Additionally, liver malondialdehyde, myeloperoxidase and catalase enzyme activity in the tissue samples were studied. Histopathological analysis was also performed.

**Results:**

No statistical difference was found between the control group and the Tropifexor group when AST, ALT and ALP values were compared. However, it was found that the Tropifexor group had statistically significant decreases in the values of GGT, total bilirubin and direct bilirubin (p < 0.05). Additionally, Tropifexor decreased the median values of malondialdehyde and myeloperoxidase, but this difference was not statistically significant compared to the control group. Finally, the Tropifexor group was statistically significant in recurring histopathological liver damage indicators (p < 0.05).

**Conclusions:**

Tropifexor reduced liver damage due to obstructive jaundice.

## Introduction

Obstructive jaundice (OJ) is the clinical condition of bile retention and jaundice which occurs due to partial or complete obstruction of intrahepatic or extrahepatic bile ducts. While choledocholithiasis and biliary structures are the leading benign causes in OJ etiology, pancreatic cancer, cholangiocarcinoma, and ampullary tumors are the leading malignant causes^1^,^2^. Bile flow into the intestine is blocked in these kinds of diseases, due to the obstruction in the bile ducts, interrupting the enterohepatic cycle. In this way, bile accumulates in the liver cells and bile ducts. As a result, increased serum levels of bile acids and bilirubin can cause liver damage. This can also cause other conditions such as cardiovascular problems, kidney failure, delay in wound healing, gastrointestinal bleeding, bacterial translocation, sepsis, multi-organ dysfunction, and death^1^,^3^.

Despite OJ being a significant health problem due to its high morbidity and mortality rates, there is no drug in routine clinical usage to prevent organ damage caused by the condition. The leading cause of liver damage in OJ is cellular damage due to bile stasis. The conventional treatment in OJ is to treat the causes of the obstruction directly. Conversely, liver damage can be prevented indirectly by reducing bile acid synthesis from the liver, thereby preventing bile retention.

The Farnesoid X receptor (FXR) is a nuclear receptor that acts as the primary regulator in the synthesis, conjugation, and transport of bile, and is highly expressed in the liver, gallbladder, intestines, and kidneys. Studies have shown that FXR agonists increase liver regeneration and have antioxidant, anti-inflammatory and hepatoprotective functions^4^. Some recent developed FXR agonist pharmacological agents have been reported to have promising results in reducing liver damage in treating non-alcoholic steatohepatitis (NASH) and primary biliary cholangitis (PBC) diseases^4^,^5^. Many other new FXR agonist pharmacological agents are currently being studied. One of them is Tropifexor (TPX), which is a potent and effective FXR agonist^5^.

The primary purpose of this study was to investigate experimentally the effects of TPX on liver injury in rats with OJ. Its effectiveness was compared with Ursodeoxycholic acid (UDCA), which is clinically used to treat cholestatic diseases and has no FXR agonist feature.

## Methods

This study was carried out at Kahramanmaras Sütçü Imam University (KSU), Medical Faculty, Experimental Research Laboratory, with the permission of the KSU Animal Experiments Local Ethics Committee (KSUTIP HADYEK), decision dated 1st Aug 2019, Session No. 2,018/07 and Decision No. 08. Also, the European Convention for the Protection of Vertebrates Used for Experimental and Other Scientific Purposes (ETS No. 123), reported by the Council of Europe, was followed in this study. Biochemical evaluations were made in the Department of Medical Biochemistry, and histopathological evaluations were performed in the Pathology Department, both being departments in the KSU Medical Faculty.

### Selection of animals and experimental conditions

In this experimental study, 40 healthy Wistar albino female rats were used. The rats were 12-14 weeks old, weighed the average of 250 ± 20 g, and each cage was populated with four rats. The animals were monitored at the average room temperature of 22 ± 2°C with suitable humidity, 12 hours of daylight and 12 hours of darkness. Standard industrial rat food was used in their diet. In the 12 hours before surgery, the rats were prevented from feeding, but they were allowed to drink water.

### Experimental groups

Five groups were created, with eight randomly selected rats in each:

- Group 1: sham (SH) was designed to compare the possible effects of anesthetic agents and the surgical process with other groups to standardize the study;
- Group 2: control (C) was designed to demonstrate the biochemical and histopathological changes caused by OJ and compare the effectiveness of the treatment groups;
- Group 3: vehicle solution (VS) was designed to differentiate the possible effects of the vehicle solution used in the oral intake of TPX, containing 0.5% methylcellulose in distilled water and 0.5% Tween 80;
- Group 4: TPX was the FXR agonist group, designed to show the effects of TPX on biochemical and histopathological parameters;
- Group 5: UDCA was designed to compare the FXR agonist’s effectiveness to UDCA since the latter cannot activate FXR when used in the treatment of cholestatic diseases.

### Anesthesia and surgical procedures

Anesthesia was employed before all surgical procedures and administered with 50 mg/kg Ketamine hydrochloride (HCL) (Ketas, Parke-Davis, Istanbul, Turkey) and 25 mg/kg Xylazine HCL (Rompun, Bayer, Istanbul, Turkey). After the anesthetic took effect, the abdomen hair of each animal was shaved. Rats were placed on the treatment table in a supine position and thoroughly disinfected by staining with 10% povidone-iodine (Poviodeks, 1,000 mL, Kimpa, Istanbul, Turkey), excluding the extremities and the upper part of the neck. Preparation for surgery was completed by covering the abdominal midline with sterile dressings.

In the SH group, the rats were subjected to laparotomy, and then the main bile duct was exposed, and the abdomen closed without additional surgery. In the other four groups, rats underwent a standard laparotomy, in which the main bile duct was isolated. The main bile duct was tied with 4/0 silk sutures, double in proximal and single in distal. Then, to prevent recanalization, the main bile duct was cut, thus creating OJ.

After the surgical procedures were completed, approximately 5 mL of saline was administered to all rats for fluid resuscitation. All groups were closed with continuous sutures in three layers using silk stitches. Feeding with standard rat food was continued for 6 hours post-operatively.

### Follow-up and drug application

After the procedure, rats were observed for clinical signs of OJ such as yellowness in the ears and mucosal surfaces, and also examined for any darkening in urine color. OJ was observable as of the third day in the groups in which the main bile duct ligation was applied.

For postoperative analgesia, 28.5 mg/kg of tramadol was used subcutaneously every 12 for 48 hours. No treatment was given to neither the SH group nor to the C group in the postoperative period. Standard food and water quantities were given to these groups for 10 days.

In the VS group, only standard food and water were given in the first three days post-operatively. After that, the carrier solution was prepared with 0.5% methylcellulose and 0.5% Tween 80 in distilled water and was given for seven days from the fourth day post-operatively with the help of an orogastric tube. Food and water intake were not restricted.

In the TPX group, only standard food and water were given in the first three days post-operatively. From the fourth day, a dose of 0.01 mg/kg/day of TPX was given in a carrier solution, administered for seven days with the assistance of an orogastric tube. The dosage of 0.01 mg/kg/dayof TPX employed in the current study is based on Tully *et al*.^5^. Food and water intake were not restricted.

In the UDCA group, only standard food and water were given in the first three days after surgery. The treatment of UDCA acid (Ursofalk^®^, Aris, Istanbul, Turkey), prepared at 25 mg/kg/day, was given with an orogastric tube for seven days from the fourth day post-operatively. Food and water intake were not restricted.

### Tissue and blood samples

On the tenth postoperative day, Ketamine 50 mg/kg/i.p.and 25 mg/kg Xylazine HCL were given to the animals per the standard laboratory conditions. Anesthesia was administered, and the abdomen was reopened from the midline. Blood was collected intra-cardiac by thoracotomy and placed into gel tubes. The livers of the animals were removed entirely and divided into two parts. For standardization, the right liver lobes were taken for histopathological examination and placed in separate containers with 10% buffered formaldehyde added. The left liver lobes were also stored under the same conditions.

### Biochemical analyses

The serum was separated by applying 5 minutes of centrifuge at 6,500 rpm to the blood samples. In the serum obtained, aspartate transaminase (AST U/L), alanine transaminase (ALT U/L), alkaline phosphatase (ALP U/L), gamma-glutamyl transferase (GGT U/L), total bilirubin (Tbil) (mg/dL) and direct bilirubin (Dbil) (mg/dL) levels were determined by Chemwell Biochemistry and the Energy Information Administration (EIA) auto-analysis device by photometric, enzymatic, and kinetic methods. As soon as the liver tissue was removed, it was placed on ice and dried with blotter paper. Before proceeding with homogenizing the tissues, 1.15% KCI was added to the tissues to provide dissolution. Tissues were homogenized at a speed of 16,000 rpm for 3 minutes. To prevent enzyme activation loss, samples were placed in an ice-filled bath. The homogenates were then centrifuged at +4°C at 14,000 rpm for 30 minutes. In the prepared homogenates, malondialdehyde (MDA), myeloperoxidase (MPO) and catalase enzyme levels in the tissue were measured as oxidant indicators and antioxidant parameters.

### Histopathological examination

For histopathological examination, tissue from the right liver lobe was fixed with a 10% buffered formalin solution. After routine tissue follow-up, 3.5 µm serial sections were taken and stained with hematoxylin-eosin (H&E). A histopathologist performed blind evaluations of the liver damage indicators with a Nikon Eclipse Ni microscope.

The following histopathological observed changes were evaluated and scored:

- 1: Sinusoidal congestion/vacuolization;
- 2: Parenchymal cell necrosis;
- 3: Bile duct proliferation.

The changes were graded by giving scores from 0 to 4 for each group. The score descriptions were as follows:

Score 0: No change;
Score 1: Very Light;
Score 2: Mild;
Score 3: Medium;
Score 4: Severe^6^.

### Statistical analysis

The package program used for data statistical analysis was Statistical Package for the Social Sciences (SPSS) 23.0. The data were expressed as the means ± standard deviation (SD). Pearson’s chi-squared analysis was used to compare variables. The tendency of variables to a normal distribution was examined using the Shapiro-Wilk test. If the data did not show normal distribution, the Kruskal-Wallis test was used to evaluate the difference between the groups. If there were a statistically significant difference between the groups, they were compared with each other using the Conover test. The statistical significance level was taken as 0.05 in all tests and stated as such when p < 0.05.

## Results

### Liver function tests

Since no OJ was formed in the SH group, AST, ALT, ALP, GGT and bilirubin values were lower than in the other groups. The median ALP value in the TPX group was 335 IU/L (min-max: 212-2,205 IU/L), while the median in the C group was 893.86 IU/L (min-max: 265-1,548 IU/L). In the TPX group, ALP values were lower than in the C group, but the difference was not statistically significant. Considering the case with the ALP values, when the TPX group and the C group were compared in terms of ALT and AST, there were no statistically significant differences.

While the GGT median value was 18.36 IU/L (min-max: 11-31 IU/L) in the TPX group, the result was 46 IU/L (min-max: 27-80 IU/L) in the C group. This value was 40.07 IU/L (min-max: 14-53 IU/L) in the VS group. The GGT value was lower in the TPX group than in the C and VS groups, and this difference was significant (p < 0.001 and p < 0.001, respectively). When the median Tbil and Dbil values were examined according to the groups, the TPX group was 6.45 (min-max: 5.17-9 mg/dL) and 6.03 mg/dL (min-max: 4.97-8.4 mg/dL), respectively. In the UDCA group, the result for Tbil was 8.86 mg/dL (min-max: 6.47-9.32 mg/dL) and for Dbil it was 8.16 mg/dL (min-max: 4.94-8.77 mg/dL). In group C, the Tbil result was 8.48 mg/dL (min-max: 7.58-10 mg/dL), and the Dbil result was 8.04 mg/dL (min-max: 7.42-9.44 mg/dL). It was observed that the bilirubin values were lower by a statistically significant amount in the TPX group compared to the UDCA and C groups (p < 0.002, p < 0.002 / p < 0.009, p < 0.001, respectively). Liver function results for all groups are shown in Table 1.

**Table 1:** Median values (min-max) of liver function tests and comparison of TPX with other groups.

	AST (U/L)	ALT (U/L)	ALP (IU/L)	GGT (IU/L)	TBil (mg/dL)	DBil (mg/dL)
SH median (min-max)	**109.5** (90-160) *	45 (37-62) *	253.5 (166-1,610)	0 (0-1) *	**0.04** (0.01-0.06) *	**0.02** (0.01-0.05) *
VS median (min-max)	401 (258-1,768)	102.5 (54-337)	494 (257-1,413)	**40.07** (14-53) *	**8.81 (3.78-** 10.36) *	**8.39** (3.46-9.89) *
C median (min-max)	354.36 (189-551)	81.5 (57-116)	893.86 (265-1,548)	46 (27-80) *	**8.48** (7.58-10) *	**8.04** (7.42-9.44) *
UDCA median (min-max)	524.15 (243.4-797.2)	79.41 (44.6-103.1)	269.64 (180-423)	21 (19-50)	**8.86** (6.47-9.32) *	**8.16** (4.94-8.77) *
TPX median (min-max)	452.57 (191-833)	78.71 (50-107)	335 (212-2,205)	18.36 (11-31)	6.45 (5.17-9)	6.03 (4.97-8.4)

SH: sham group; VS: vehicle solution group; C: control group; UDCA: Ursodeoxycholic acid group; TPX: Tropifexor group; AST: aspartate transaminase; ALT: alanine transaminase; ALP: alkaline phosphatase; GGT: gamma-glutamyl transferase; Tbil: total bilirubin; Dbil: direct bilirubin;

* statistically significant different TPX *vs*. other groups (p < 0.05).

### Liver tissue oxidant-antioxidant parameters

In the TPX group, the median tissue MDA values were 1.47 nmol/mg (min-max: 0.64-2.66 nmol/mg), and the median tissue MPO values were 90.03 U/g (min-max: 75.93-107 U/g). In group C, the MDA median values were 1.9 nmol/mg (min-max: 0.92-3.03 nmol/mg), and the MPO median values were 97.25 U/g (min-max: 72.22-111.11 U/g). Although these oxidant parameters were lower in the TPX group than in the C group, the observed decrease was not statistically significant (Table 2). There was no statistically significant difference between the tissue catalase value of the TPX group and the other groups.

**Table 2:** Liver tissue oxidant-antioxidant median (min-max) parameters and comparison of TPX with other groups.

	Catalase (U/mg protein)	MDA (nml/mg protein)	MPO (U/g protein
SH median (min-max)	231.47 (54.59-315.79)	2.66 (0.85-3.27) *	98.39 (86.8-113.02)
VS median (min-max)	120.62 (46.61-285.38)	2.65 (0.64-4.03)	108.31 (92.35-160.5) *
C median (min-max)	88.97 (50.86-159.84)	1.9 (0.92-3.03)	97.25 (72.22-111.11)
UDCA median (min-max)	119.38 (41.91-166)	2.56 (1.72-3.76) *	87.09 (69.25-107.34)
TPX median (min-max)	98.87 (75.24-130.83)	1.47 (0.64-2.66)	90.03 (75.93-107)

SH: sham group; VS: vehicle solution group; C: control group; UDCA: Ursodeoxycholic acid group; TPX: Tropifexor group; MDA: malondialdehyde, MPO: myeloperoxidase;

* statistically significant different TPX *vs*. other groups (p < 0.05).

### Histopathology

**Figure 1:**
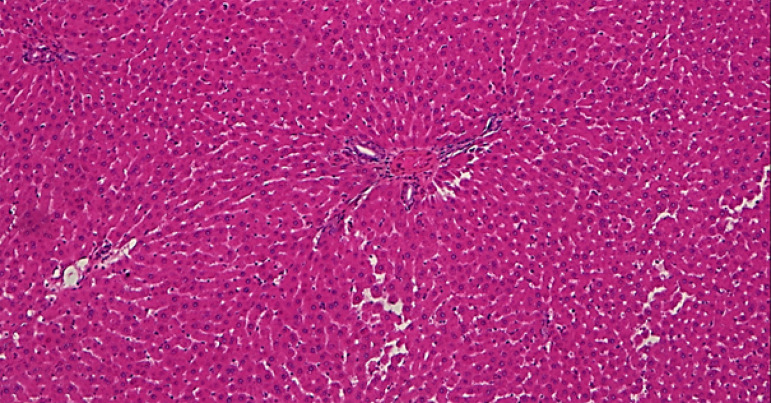
Normal histopathological view from the sham group (hematoxylin-eosin x200).

**Figure 2:**
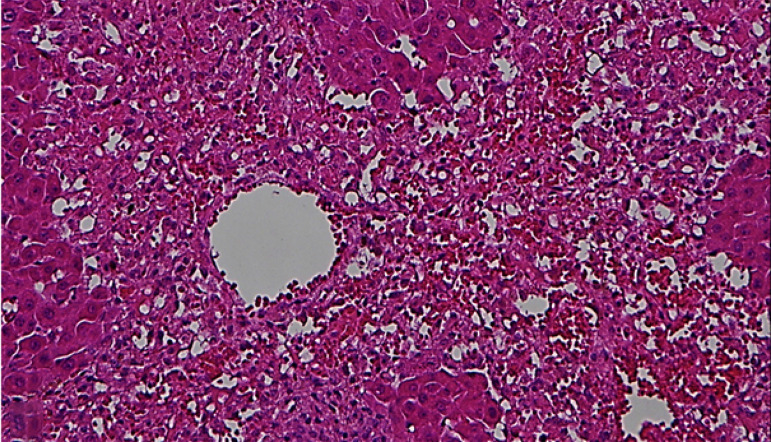
Severe areas of necrosis in the liver from the control group (hematoxylin-eosin x200)

**Figure 3:**
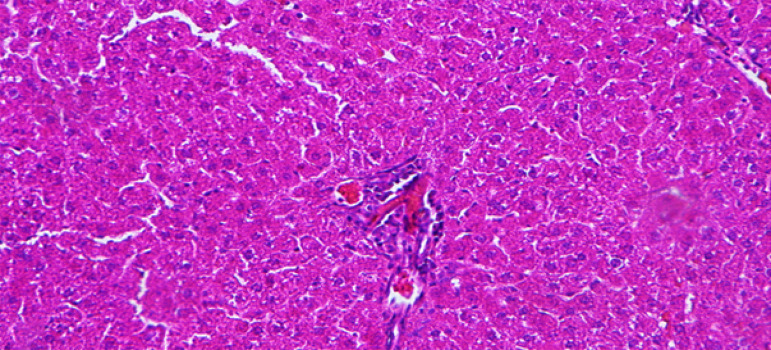
Moderate signs of sinusoid congestion/vacuolization and necrosis in parenchymal cells from the Tropifexor group (hematoxylin-eosin x200).

It was observed that there were normal liver findings in the SH group (Fig. 1). Sinusoidal congestion/vacuolization, necrosis in the parenchymal cells, and bile duct proliferation were all moderately more severe compared to the C and VS groups. The difference in the severity was statistically significant (Figs. 2 and 3). However, there was no statistically significant difference between the TPX and UDCA groups according to the distribution of histopathological findings (p > 0.05) (Table 3).

**Table 3:** Histopathological changes and comparison of TPX with other groups.

Score (0-4)	Sinusoidal congestion / vacuolization	Parenchymal cell necrosis	Bile duct proliferation	(n)
SH	0, 0, 0, 1, 0, 1, 1, 0	0, 0, 0, 0, 0, 0, 0, 1	0, 1, 1, 1, 0, 0, 1, 0	8
VS	4, 3, 2, 3, 2, 3, 4, 2 *	3, 4, 2, 3, 3, 2, 3, 4 *	4, 3, 3, 4, 2, 3, 4, 2 *	8
C	2, 3, 2, 3, 4, 3, 2, 3 *	2, 4, 4, 3, 2, 3, 3, 3 *	2, 3, 3, 4, 2, 4, 3, 3 *	8
UDCA	2, 2, 2, 1, 1, 1, 1, 1	2, 1, 1, 2, 1, 1, 1, 2	1, 1, 1, 2, 2, 1, 2, 2	8
TPX	2, 1, 2, 1, 1, 1, 2, 2	2, 1, 1, 1, 2, 1, 1, 2	1, 2, 2, 1, 1, 1, 2, 2	8

SH: sham group; VS: vehicle solution group; C: control group; UDCA: Ursodeoxycholic acid group; TPX: Tropifexor group;

* statistically significant different TPX *vs*. other groups (p < 0.05).

## Discussion

Two main factors come to the fore in the physiopathology of liver injury occurring in OJ. The first one is the hepatotoxic effects caused by bile salts and bilirubin due to bile stasis. The other factor is the deterioration in the balance of oxidative and antioxidant systems in the liver and increased lipid peroxidation^7^. This damage can be prevented with a drug that can reduce bile acid synthesis and exhibit antioxidant properties, targeting the elimination of these OJ related causes of liver damage.

In the biochemical findings of patients with OJ, AST, ALT, ALP, GGT and bilirubin values increase due to the toxic effect of cholestasis^8^. There are elevated biochemical AST, ALT, ALP and GGT values in NASH disease, which is associated with a fatty liver, inflammation, hepatocyte swelling, and fibrosis. The same occurs in PBC disease, an autoimmune cholestatic disease. The level of liver enzymes is a significant predictor in evaluating the effectiveness of the treatments given in NASH and PBC diseases. UDCA is the first and most preferred drug used in treating PBC patients, effectively reducing liver enzymes and inhibiting liver damage.

However, some PBC patients do not respond to treatment with UDCA, and in those cases the disease continues to show progression. Recently, studies have been carried out with various FXR agonists to treat these patient groups which do not respond to treatments^9^. FXR is an NR and decreases bile acid synthesis and absorption while increasing excretion. Thus, it plays a key role in bile acid metabolism. Moreover, FXR activation has been shown to increase liver regeneration and to have anti-inflammatory and antioxidant effects^10^,^11^.

In a double-blind, placebo-controlled phase-3 study performed by Nevens *et al*.^12^, it was observed that serum ALP and Tbil levels decreased. This was especially the case with PBC patients who did not respond to UDCA and who were in the group receiving obeticholic acid (OCA), an FXR agonist. The researchers also reported that OCA was effective in healing the disease by decreasing AST, ALT, and GGT serum levels. In the NASH murine model study, Zhang *et al*.^13^ showed that WAY-362450, an FXR agonist, decreased AST function and ALT values. According to the published preliminary results of the phase-2 placebo-controlled study with Cilofexor (an FXR agonist) in PBC patients, AST, ALT, GGT and ALP serum levels were effectively reduced^14^.

In the biochemical results of the current study, no statistically significant difference was found in the serum levels of AST, ALT and ALP between the TPX group and the other ones. According to the authors’ view, this is due to the 7-day TPX treatment duration in this study. Eloy *et al*.^10^, in their study with the NASH animal model, stated decreased AST and ALT values after the second week of TPX treatment. Therefore, it is hypothesized that, if the TPX treatment period were extended, similar results would be achieved in this study.

The serum levels of GGT, Tbil, and Dbil levels in this study’s TPX group were lower by a statistically significant amount compared to those of the control group. Nevens *et al*.^12^ and Liu *et al*.^15^ also showed that FXR agonists effectively lows ALP level. Additionally, Liu *et al*.^15^ reported that FXR agonist GW4064 decreased bilirubin values, but this was not statistically significant compared to taurodeoxycholic acid. However, in the current study the TPX group’s bilirubin values were statistically and significantly lower than both the UDCA and control groups. This improvement in cholestasis parameters is due to TPX (an FXR agonist) reducing bile synthesis and the accompanying anti-inflammatory activity. The achievement of lower serum levels of GGT, Tbil, and Dbil due to TPX is an important indicator for its future application in OJ.

Increased intraductal pressure caused by bile retention in OJ and cellular swelling in hepatocytes creates an ischemic injury. Subsequently, an increase in the production of superoxide radicals (SOR) occurs, and excessive SOR formation leads to polymorphonuclear leukocyte (PMNL) activation. In these cases, mediators released by PMNL in the form of a cycle increase SOR production even more. Accordingly, oxidative stress and an increase in lipid peroxidation induced by SOR cause damage to the liver and biliary tract^7^,^16^. Under normal conditions, SOR is rendered ineffective after its removal by antioxidant mechanisms. However, increasing SOR and decreasing antioxidant activity in OJ, the balance between oxidative and antioxidative systems deteriorates. As a result, antioxidants remain inadequate and facilitate the occurrence of liver damage^7^.

Tsuji *et al*.^17^ reported that PMNL activation and cytokine and SOR production from neutrophils increased more in OJ rats than in normal rats. Alturfan *et al*.^18^ also reported that MPO and MDA levels, which are indicators of PMNL activation, increased in rats with OJ, which was consistent with liver damage. Livero *et al*.^19^ gave mice 6-alfa-ethil-chenodeoxycholic acid (ECDCA) (obeticholic acid:OCA), which is an FXR agonist, after generating hepatic steatosis with ethanol in them. They showed that, in the group given 6-ECDCA, catalase and SOD activity increased, and oxidative stress decreased. Eloy *et al*.^10^ in a study in which they investigated the antioxidant activity of TPX reported that it up-regulates glutathione S-transferase a4 and glutathione S-transferase genes, which are dose-dependent antioxidant genes.

The current study evaluated liver tissue MDA and MPO levels as oxidative markers and liver tissue catalase levels as antioxidant markers. It was observed that the mean tissue MDA value of the TPX group was lower than in all the other groups. However, this result was not statistically significant when compared to the control group. The efficiency of TPX was not detected in other oxidative markers studied. It is possible this observation was related to the dose and duration of TPX used in this study. Tully *et al*.^5^, in their paper regarding the discovery of TPX, showed that, as the dose of TPX increased, its effectiveness increased. Therefore, the current authors’ view is that, increasing the TPX dose and duration of treatment, better outcomes would be achieved on oxidative and antioxidative parameters.

Liu *et al*.^15^ showed that bile duct proliferation, parenchymal necrosis and inflammatory cell infiltration were increased in OJ rats and that these findings regressed significantly in the group given the FXR agonist GW4064. Hu *et al*.^20^ reported that, histopathologically, the vacuolization and inflammation findings were significantly reduced in the group given INT-767 (an FXR agonist).

In the present study, the measurement of sinusoidal congestion/vacuolization, presence of necrosis in parenchymal cells and bile duct proliferation were evaluated as histopathological markers of liver damage. It was found that the difference between the TPX group and the C and VS groups were found to be statistically significant in the improvement of histopathologic liver damage markers. This is an indication of TPX’s potential effectiveness in preventing liver damage in OJ. However, no statistically significant difference was found between the TPX group and the UDCA group. This may show that TPX and UDCA have a similar level of effect after all. UDCA is a commercial drug with clinical use. The fact that TPX appears as effective as UDCA demonstrates that this issue is worth investigating. Consistent with the literature on the topic, it must be emphasized that TPX is a promising agent and that, as an FXR agonist, it is effective in reducing liver damage histopathologically in rats with OJ.

As with all studies, this one comes with some limitations. Some weaknesses include the low sample size and the application of TPX in a narrow dose range. The short duration of treatment of TPX can also be considered a limitation. Alternatively, if evaluated together with the literature on reducing liver damage due to OJ, it can be said that TPX is a potentially effective agent. However, additional studies are needed in this regard before clinical usage begins.

## Conclusion

This study showed that TPX is effective in reducing liver damage in rats with OJ. TPX may be a viable step forward in the treatment of OJ, reducing morbidity and mortality, although additional experimental and clinical studies are needed.
